# Single-copy sensitive, field-deployable, and simultaneous dual-gene detection of SARS-CoV-2 RNA via modified RT–RPA

**DOI:** 10.1038/s41421-020-0175-x

**Published:** 2020-05-28

**Authors:** Simin Xia, Xi Chen

**Affiliations:** The HIT Center for Life Sciences, Harbin Institute of Technology, Xidazhi Street 92, Harbin, Heilongjiang 150001, China

**Keywords:** Transcription, Genomic analysis

Dear Editor,

Since the end of 2019, the world is suffering an outbreak of COVID-19 pneumonia caused by SARS-CoV-2 (2019-nCoV)^1^ with over 84,000 infections in China and over 3,000,000 infections outside China worldwide (as of April 28th, 2020). The whole genome of SARS-CoV-2 was sequenced and then released to the public on January 5th, 2020^2^, which served as the basis for nucleic acid-based diagnostics such as reverse transcription–polymerase chain reaction (RT–PCR)^3,4^. Since human-to-human transmission has been confirmed^5,6^, field-deployable detection methods against SARS-CoV-2 are highly required (Supplementary Fig. S1). In addition, detection sensitivity is important for early-stage diagnostics and to reduce false-negative results. While RT–PCR is widely used to detect viral RNA, it is not readily field-deployable because of the high cost of real-time PCR machine and the expertise required to perform the analysis. Antibody-based diagnostics are usually field-deployable, but it takes weeks or months to produce high-titer antibodies; additionally, they are usually not as sensitive and specific as nucleic acid-based approaches^7^. Gene editing has also recently been introduced for the detection of RNA, which requires multiple reaction steps^8^.

In this regard, we herein introduced an ultrasensitive field-deployable approach to detect SARS-CoV-2 gene by applying reverse transcription–enzymatic recombinase amplification (RT–ERA) (Fig. 1a). ERA is a modified version of recombinase polymerase amplification (RPA)^9^ introduced by GenDx Biotech, belonging to isothermal nucleic acid amplification techniques that can be carried out at constant temperatures without the need for thermocycles^10,11^. In our one-pot multienzyme RT–ERA reaction system, RNase inhibitor, reverse transcriptase, recombinase, polymerase, single-stranded DNA-binding protein, creatine kinase, and nuclease are present to enable the detection of RNA. A fragment of N gene and a fragment of S gene within the entire viral genome were selected for detection (Supplementary Fig. S2a). We subsequently prepared high-quality RNA fragments as the standards via in vitro transcription using T7 RNA polymerase (Supplementary Fig. S2b, c).

**Fig. 1:**
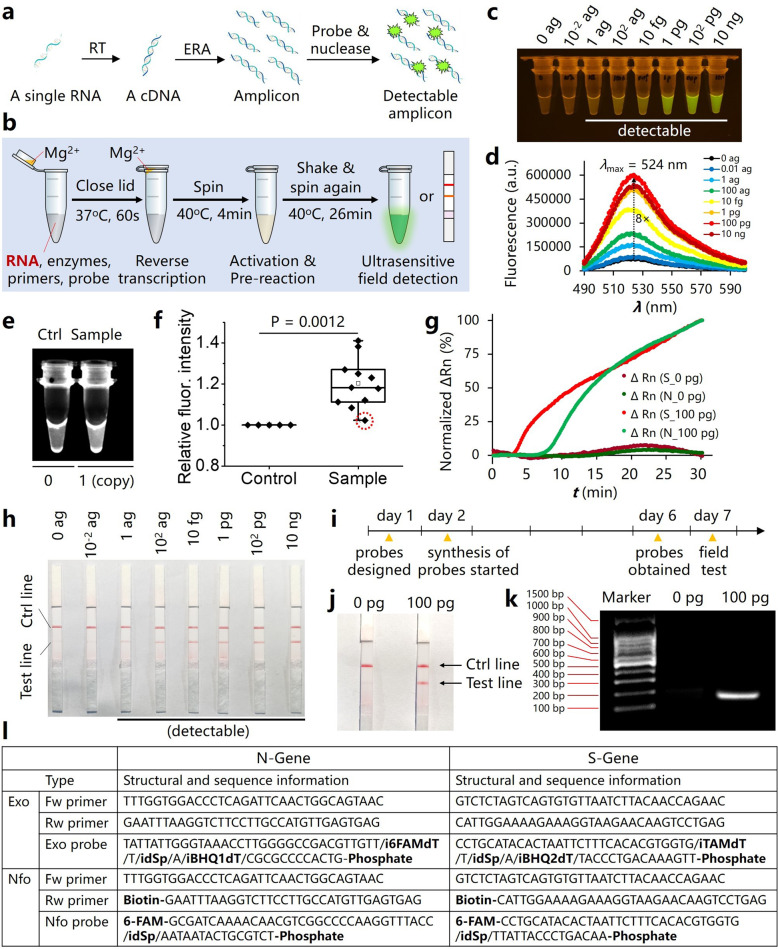
Single-copy sensitive, field-deployable, and simultaneous dual-gene detection of SARS-CoV-2 RNA via modified RT–RPA. **a** The basic principle of RT–ERA. **b** Schematic view of the WEPEAR protocol. **c** Fluorescence detection using a blue light imager. **d** Quantitative analysis of the fluorescence enhancement (*λ*_ex_ = 455 nm). **e** One representative example in the single-copy S-RNA detection experiment. **f** Statistical analysis where Student’s *t* test was used. **g** Time course of simultaneous dual-gene detection where ΔRn is plotted against time; for the detection of the N gene: *λ*_ex_ = 470 ± 15 nm, *λ*_em_ = 520 ± 15 nm, whereas for the detection of the S gene: *λ*_ex_ = 550 ± 11 nm, *λ*_em_ = 586 ± 10 nm. **h** Detection of the N gene using nfo probe and LF strips. **i** Key time points in designing an nfo probe and primers for field detection of the S gene. **j** Detection of the S gene using the nfo probe and LF strips. **k** Agarose gel electrophoresis analysis of the nfo RT–ERA reaction. **l** Sequence and structural information of the probes and primers used in this study.

We first introduced exo FRET (fluorescence resonance energy transfer) probe for fluorogenic detection of SARS-CoV-2 N gene (Fig. 1l, upper left). We designed an exo forward and reward primer pair for specific amplification of the RNA specimen via RT–ERA. BLAST analysis revealed that this primer pair indeed specifically detects SARS-CoV-2. Meanwhile, an exo FRET probe was designed to specifically detect the amplicon (Supplementary Fig. S3). We found that 100 units of exonuclease III serve as an optimal level to trigger the green fluorescence increase (Supplementary Fig. S4). In a similar way, we designed the respective primers and probes for the detection of the S gene (Fig. 1l, upper right). Regarding the exo probe for the S gene, we employed the red-emissive TAM fluorophore so that it may pair with the green-emissive FAM fluorophore for simultaneous dual-gene detection.

We also introduced a WEPEAR (**w**hole-course **e**ncapsulated **p**rocedure for **e**xponential **a**mplification from **R**NA) procedure for “sample-in, results-out” one-pot detection of RNA in a sensitive and reliable way (Fig. 1b). In the WEPEAR protocol, the RNA sample is added into a reaction mixture containing all necessary components for both RT and ERA, aside from the ERA activator—Mg^2+^, while 2 µl of Mg^2+^ is loaded inside the lid of the reaction vial. Mg^2+^ liquid will still stay inside the lid after gently closing the lid due to surface tension. The reaction vial is first placed in a 37 °C water bath for 60 s to allow only RT to occur. After spinning, Mg^2+^ activator is mixed and triggers the ERA reaction. The reaction vial is heated to 40 °C for 4 min to allow the initial ERA reaction to proceed. Finally, shake and spin the vial again (essential to enhance detection sensitivity since the reaction mixture is highly viscous), and let the reaction proceed for additional 26 min at 40 °C. Finally, the reaction product can be simply detected via green fluorescence excited by blue light. During the entire course, the reaction proceeds through four steps: RT, ERA activation, pre-reaction, and final reaction, to give fluorescence readout without uncovering the lid at all. Hence, it greatly reduces any possible contaminations (e.g., via aerosol), and ensures the reliability of this approach to detect trace amounts of RNA.

We found that the exo FRET probe in combination with WEPEAR gave consistently ultrahigh sensitivity that as low as 1 ag (10^−18^ g) of RNA can be detected (Fig. 1c, d). On account of the M.W. of the RNA (1,47,655 Da, ~21 µl of sample), less than 0.32 aM (i.e., <0.2 copy/µl) of RNA could be detected, suggesting that this is an ultrasensitive detection method. We further confirmed this ultrasensitivity by repeating the detection multiple times combined with statistical analysis (Supplementary Fig. S5). We also plotted the fluorescence intensity of each reaction solution against the mass of RNA, and found that a half-effect mass of the RNA sample to be detected is around 8 fg (Supplementary Fig. S6). Since this method only requires a mini-blue light imaging plate, or simply a mini-blue light flashlight (few dollars) plus an orange-colored plastic film, this detection method is field-deployable and can be applied to grassroot clinics. Meanwhile, we demonstrated that the exo FRET probe can also be implemented in an advanced real-time PCR station that allows real-time detection of the fluorescence enhancement (Supplementary Fig. S7). The maximal reaction intensity is reached after 25 min, suggesting that 30 min of the total reaction time in our protocol is a suitable parameter using the exo FRET probe.

With the exo probe and primers designed for the S gene, we achieved even better sensitivity that 1 ag of the S-gene RNA gave much higher fluorescence compared with control (0-ag RNA) (Supplementary Fig. S8), suggesting that the exo probe for the S gene was superiorly designed. Hence, we were motivated to check if single-copy sensitivity, the highest possible sensitivity, could be achieved. We modified the WEPEAR protocol a bit that the reaction vial was shaken and spun at 3, 6, and 9 min during the reaction course. We prepared an extremely diluted RNA solution at 1 copy/µl (~0.25 ag/ul) as the standard. We ran five times of independent experiments, and in each experiment, one or three sample reactions (each 1-copy RNA) were included in addition to a blank control (0-copy RNA). After the RT–ERA reaction, 10 out of 11 reaction vials showed brighter fluorescence compared with their respective blank controls (Fig. 1e, f). There was one vial that did not show clearly brighter fluorescence than control (Fig. 1f, within the red dashed circle). This might be due to the absence of an RNA molecule in the reaction solution. Indeed, there is a chance that a single molecule may not be taken out from the 1 copy/µl stock solution in 1 µl using a pipette. We confirmed that the single-copy detection limit is highly reliable using Student’s *t* test (*P* = 0.0012). We also demonstrated the detection specificity of these exo probes and primers for SARS-CoV-2 over other highly similar coronaviruses such as SARS-CoV (Supplementary Fig. S9).

In ultrasensitive detection methods, it would be great if two genes could be simultaneously detected to ensure reliability, which will reduce the chance of false positives. Therefore, we paired the exo probes and primers for the N gene (green) and S gene (red) in one reaction, and used the real-time PCR machine to record the time courses. After optimization of parameters, we found that 100 nM of each primer and 30 nM of each probe worked well. Both N- and S genes were detected simultaneously with high signal-to-noise ratios (Fig. 1g). The results also suggested that the exo probe for the S gene is more sensitive than that for the N gene because the fluorescence intensity for the S gene starts to undergo an exponential increase at ~3 min, which is earlier than at ~7 min for the N gene.

In order to make the detection approach simpler, or even can be conducted at home, we were motivated to design the so-called nfo-affinity probe system plus lateral flow (LF) strips for RNA detection (Fig. 1h). We designed a pair of nfo forward and reward primers to amplify an amplicon within the N gene. Besides, we designed a nfo-affinity probe for the detection of the N gene (Fig. 1l, lower left) according to the general design principle (Supplementary Fig. S10). We were delighted to find that this detection approach is also ultrasensitive that as low as 1 ag of the N-gene RNA can be detected visually (Fig. 1h). We further validated this ultrasensitivity by repeating the lateral flow assay several times (Supplementary Fig. S11a), and purified the amplicon from each reaction to confirm the presence of the amplicon with a M.W. of around 150 bp via agarose gel electrophoresis (Supplementary Fig. S11b).

In the nfo-affinity detection approach, a temperature-controlled water bath or a heating block may still be needed, which are usually inexpensive, but may not be always available. Therefore, we checked that the nfo-affinity detection approach could be performed by simply using a thermos cup that is easily available. By gradually adding hot water into a thermos cup containing room-temperature water with stirring, it is easier than expected to make 40 °C water. Then a control reaction vial (0-pg RNA) and a sample vial (100-pg RNA) were put into the cup via a float followed by covering the thermos cup by a foam (Supplementary Fig. S12a). In multiple trials, the temperature only dropped 1 °C in 30 min. The RT–ERA reaction mixture was diluted and then tested by gold LF strips. We found that SARS-CoV-2 RNA could be easily detected using this household setup (Supplementary Fig. S12b). Motivated by the ultrahigh sensitivity of this nfo- affinity detection approach, we mixed SARS-CoV-2 RNA gene directly with diluted throat swab without extra sample processing nor RNA purification. Delightfully, SARS-CoV-2 RNA in throat swab could still be clearly detected by comparing with blank control, suggesting that even the sample-processing step could be skipped using the nfo-affinity detection approach (Supplementary Fig. S12c). These experiments highlighted that the nfo-affinity probe could be applied not only in a field-deployable way, but also in a household fashion.

Finally, we designed the nfo probe and primers for S-gene detection (Fig. 1l, lower right). In this regard, we showed that such a field-deployable detection could be introduced in a few days, which would meet the urgent need upon a viral outbreak in the future. The nfo probe and primers for the S gene were designed on day 1, subjected to synthesis on day 2, obtained on day 6, and decent field-detection results using lateral flow strips were achieved on day 7 (Fig. 1i, j). We confirmed that the bifunctionalized amplicon is present in the RT–ERA reaction solution via agarose gel electrophoresis (Fig. 1k). We further showed the detection specificity of these nfo probes and primers for SARS-CoV-2 over other similar coronaviruses such as MERS-CoV and SARS-CoV (Supplementary Fig. S13).

In summary, we introduced single-copy-sensitive field-detection approaches against SARS-CoV-2 RNA genes. The detection limit reaches 1 copy (0.05 copy/µl, on account of >20 µl of sample volume); hence, it facilitates the development of digital RT–RPA for quantitative RNA detection where single-copy sensitivity is required^12^. In order to match the ultrasensitivity of these detection approaches, we introduced a WEPEAR procedure for “sample-in, results-out” detection of RNA in one pot. By pairing the exo probe for N and S genes, we achieved simultaneous detection of two genes in one reaction by applying modified parameters. More importantly, these nucleic acid detection approaches are field- or even household-deployable. The exo FRET probe detects the RNA gene via fluorescence enhancement, while the nfo-affinity probe detects the RNA gene using LF strips. We hope that the exo FRET probe, nfo-affinity probe, and the respective primers we designed here could not only facilitate the rapid, sensitive, and field detection of SARS-CoV-2, but also be helpful to guide the design of new primers and probes when SARS-CoV-2 mutates or a next RNA virus outbreak occurs.

## Supplementary information

Supplementary information

## Supplementary information

**Supplementary Information** accompanies the paper at (10.1038/s41421-020-0175-x).
