# Supplementary material for: Single-copy sensitive, field-deployable, and simultaneous dual-gene detection of SARS-CoV-2 RNA via modified RT–RPA
Source: Cell Discov. 2020 May 28;6:37. doi: 10.1038/s41421-020-0175-x (PMC7253471; doi:10.1038/s41421-020-0175-x)
Supplement: Supplementary file 1 — Supplementary information [file 41421_2020_175_MOESM1_ESM.pdf]

Supporting Information

1. T7 transcription to prepare viral RNA fragments of different coronaviruses

For the preparation of coronal viral RNA gene fragments, the respective deoxynucleotide sequence was subjected to customer gene synthesis from LoGenBio (Shanghai, China), cloned into pET-28a(+) vector with NdeI and HindIII restriction cleavage site. Afterwards, the vectors were subjected to linearization cut by HindIII restriction enzyme. Briefly, in a 50μl reaction system, 5μl of HindIII and 5μl of 10x CutSmart buffer were added into 40μl of vector solution on ice. Afterwards, the reaction mixture was heated to 37°C for 4 hours to overnight to allow complete linearization, as confirmed by agarose gel electrophoresis analysis. The linearized plasmid was separated via agarose gel electrophoresis and purified using FastPure Gel DNA Extraction Mini Kit from Vazyme Biotech co., Ltd. (# DC301).

The in vitro transcription was performed using T7 high yield transcription kit from Vazyme Biotech co. Ltd. (#TR101-01). In a 20μl reaction system, 8μl of the above prepared linearized plasmid was added with 2μl of 10x reaction buffer, 2μl of ATP solution, 2μl of GTP solution, 2μl of UTP solution, 2μl of CTP solution and 2μl of T7 RNA polymerase Mix that contains T7 RNA polymerase and other necessary components. The reaction is proceeded at 37°C for 4h to overnight. Afterwards, 1μl of DNase A (included in the kit) was added into the reaction vial and the vial was incubated at 37°C for additional 15min in order to digest the plasmid template. The 20μl of reaction solution was diluted to 150μl, and purified using Express RNA Purification Kit from GenDx Biotech Co., Ltd (# NR202-50T). Agarose gel electrophoresis analysis revealed the formation of a single band RNA with M.W. between 400 to 500 bp.

2. RT-ERA protocol for exo FRET probe detection

The RT-ERA reaction was performed using basic RT-ERA reaction kit from GenDx Biotech Co., Ltd. (# KS102). In a 50μl of reaction system, 2.5μl of exo forward primer (10 μM), 2.5μl of exo reward primer (10 μM), 0.75μl of exo FRET probe (10 μM), 1μl of 5 U/μl of murine RNase inhibitor (Vazyme, # R301-01), 1μl of RNA sample, 20μl of DI-H_2O, and 0.5μl of 200U/μl exonuclease III (Exo III, thermo scientific, # EN0191) were added into 20 ul of DA solution (dissolving buffer). The resultant 48μl of reaction solution was added into the PCR tube containing dry power that includes all necessary enzymes and ingredients for RT-ERA to occur. Gently pipette to mix the reaction mixture. Meanwhile, 2μl of $Mg(OAc)_2$ solution as the ERA activator was loaded into the lid of the PCR tube, then close the lid gently. Afterwards, the reaction vial was incubated in a 37°C water bath for 60s to allow solely transcription to take place. Afterwards, spin the tube to mix the Mg^{2+} activator with the RT-ERA reaction mixture, shake and spin again to collect all liquids to the bottom of the PCR tube. The PCR tube was heated at 40°C for 4min using a heating block or PCR machine to allow pre-reaction. Finally, the PCR tube was shaken and then spin again, and incubated at 40°C for another 26min to complete the RT-ERA reaction. A blank control without adding any RNA but instead pure water needs to be included for each time of detection in order to exclude any possible false positive results.

The fluorescence signal can be detected by putting the PCR tube on top of a mini blue-light plate and capture the fluorescence image a smartphone camera. Alternatively, the fluorescence intensity can be quantified using Molecular Devices SpectraMax i3x with a 96-well plate . For real time determination of

the fluorescence enhancement, Applied Biosystem QuantStudio 6Flex real-time PCR machine was used in which PCR tubes with flat and transparent lids must be used.

3. RT-ERA protocol for nfo affinity probe detection

The procedure is a bit different from the protocol for exo FRET probe detection. The RT-ERA reaction was also performed using the basic RT-ERA reaction kit from GenDx Biotech Co., Ltd. (# KS102). In a 50ul reaction system, 2µl of nfo forward primer (10 µM), 2µl of nfo reward primer (10 µM), 0.6 µl of nfo probe (10µM), 1µl of 5U/ul of murine RNase inhibitor (Vazyme, # R301-01), 1µl of RNA sample, 21ul of DI-H_2O, and 1µl of 10U/µl endonuclease IV III (NEB, # M0304S) were added into 20µl of DA solution (dissolving buffer). The resultant 48µl of reaction solution was added into the PCR tube containing dry power that includes all necessary enzymes and ingredients for RT-ERA to occur. Gently pipette to mix the reaction mixture. On the other hand, 2µl of $Mg(OAc)_2$ solution as the ERA activator was loaded into the lid of the PCR tube, then close the lid gently. Afterwards, the reaction vial was incubated in a 37^oC water bath for 60s to allow only the transcription to take place. Afterwards, spin the tube to mix the Mg^{2+} activator with the RT-ERA reaction mixture, shake and spin again to collect all liquids to the bottom of the PCR tube. The PCR tube was heated at 40^oC for 4min using a heating block or PCR machine or water wall inside a thermos cup to allow pre-reaction. Finally, the PCR tube was shaken and then spin again, and incubated at 40^oC for another 26min to complete the RT-ERA reaction. A blank control without adding any RNA but pure water needs to be included for each time of detection in order to exclude any possible false positive results.

For the detection using lateral flow (LF) strips, HybriDetect LF strips paper from Amplification Future (# WLF8201) was used. Briefly, 10µl of nfo reaction solution was diluted in to 200µl by DI-H_2O in a 1.5ml centrifuge tube. The sample pad end of the a HybriDetect LF strip was dipped into the diluted reaction solution and after 2-3min, the test line will become red which indicates that SARS-CoV-2 virus gene is detected.

4. Agarose gel electrophoresis

For the analysis of plasmids and linearized vectors, 1 % agarose (0.4 g of agarose dissolved in 40 ml of TAE buffer) was used while DL5000 DNA marker (Vazyme, # MD102) was used as the ladder. For the analysis of short DNA amplicon or RNA fragments, 2 % agarose gel (0.8 g of agarose dissolved in 40 ml of TAE buffer) was used while 100 bp DNA ladder (Vazyme, # MD104) was used as the ladder. In both cases, the electrophoresis was performed under 200 V constant voltage for 15 min in 1x TAE buffer.

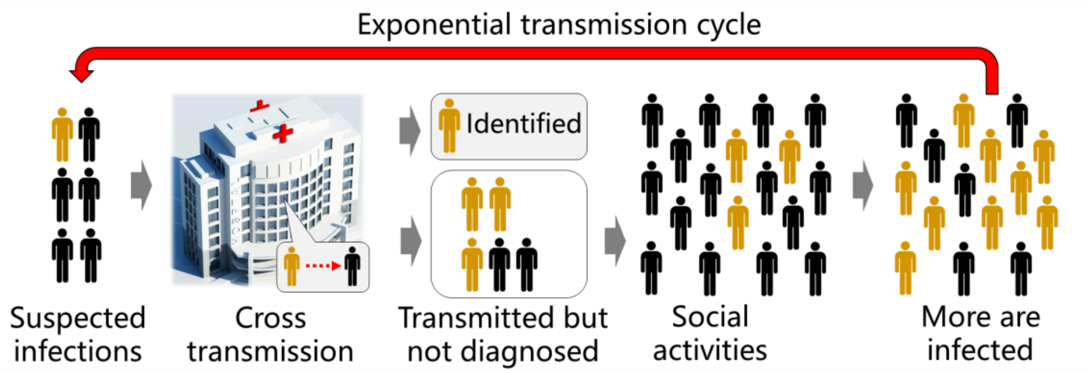

Figure S1. Due to cross-transmission of SARS-CoV-2, sensitive and field-deployable nucleic acid detection methods are needed.

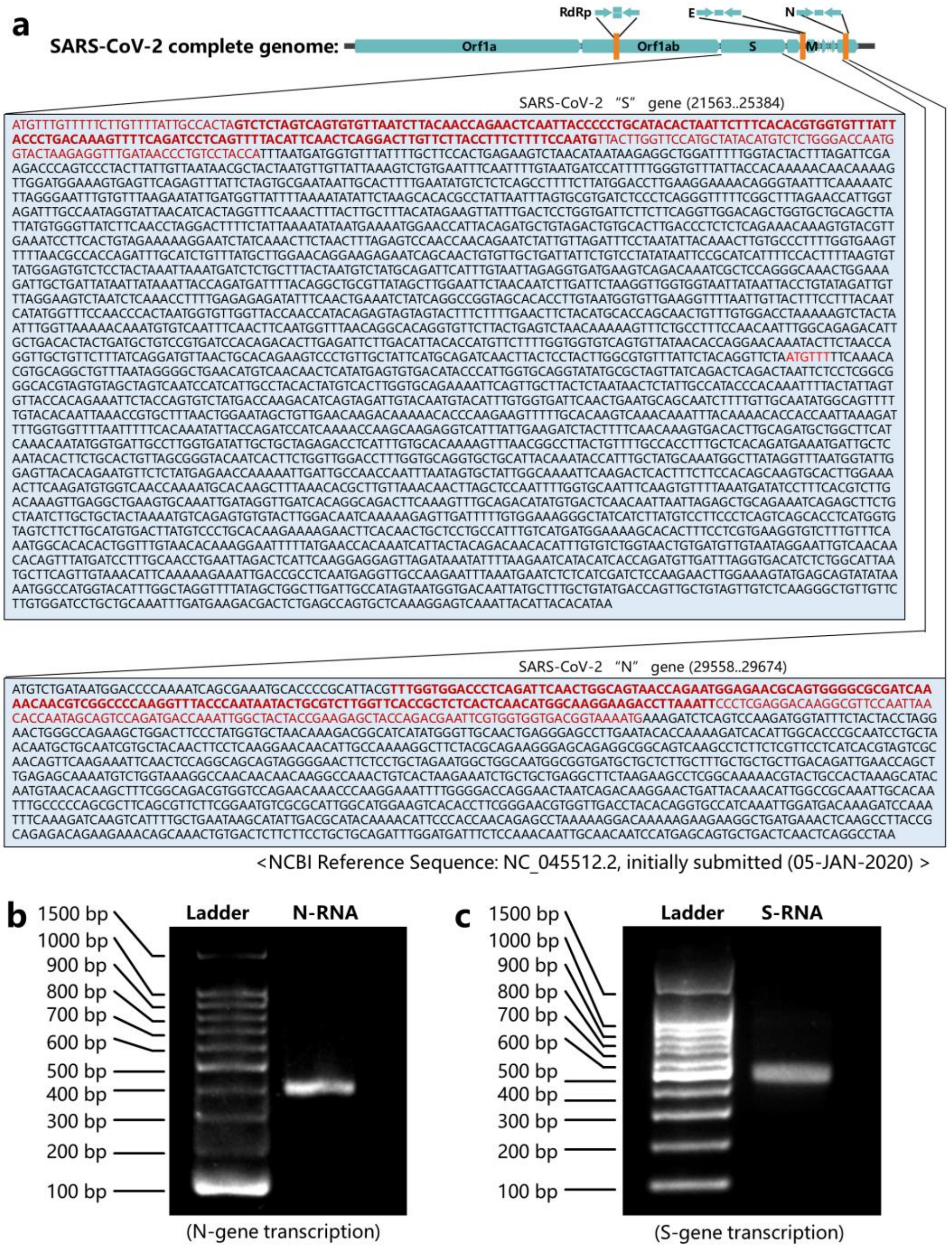

Figure S2. a, Schematic view of the complete SARS-CoV-2 genome and the full sequence of the S-and N-genes. Note that the sequence region in dark red are characteristic regions to be detected, and that all U are denoted as T. **b,c**, The gene sequences highlighted in dark red in A are synthesized, cloned into a pET-28a(+) vector, linearized by HindIII restriction cleavage, and transcribed to RNA using T7 polymerase to give N- and S-RNA genes as targets for subsequent assay development. Single band high purity RNAs are obtained.

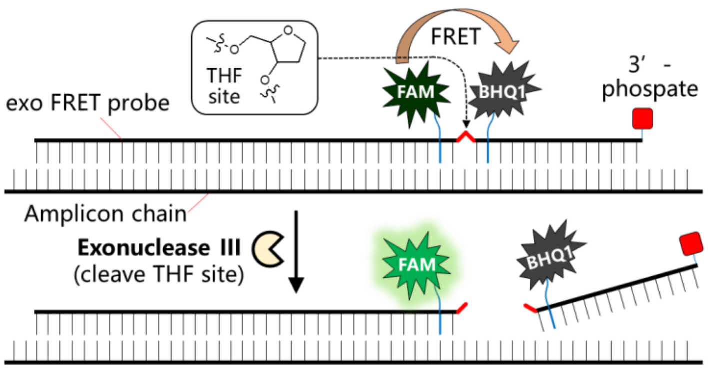

Figure S3. General guidelines for the design of an exo probe. Exo FRET probe features 33bp bases at its 5'-side, 12bp bases at its 3'-side, a phosphate blocking group at its 3'-side so that this probe cannot act as a primer. This FRET probe specifically hybridizes with one chain of the amplicon. Additionally, this probe features a 6-FAM modified T-residue (i6FAMdT) and a BHQ1 quencher modified T-residue (iBHQ1dT) in close proximity separated by 3 bases; thus, the fluorescence of FAM is temporally quenched by its FRET quencher—BHQ1. Finally, the exo FRET probe features an abasic tetrahydrofuran (THF) residue (idSp) between the FRET pair. THF residue is stable against Exo III when the FRET probe is in a single-stranded state, but will be cleaved by Exo III when the probe hybridizes with one chain of the amplicon in a double-stranded state. Then BHQ1 releases and the fluorescence of FAM moiety is restored.

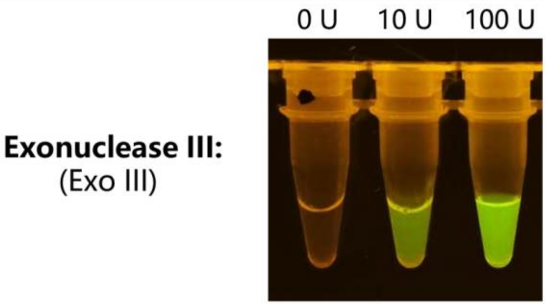

Figure S4. Evaluation of the optimal amount of Exo III enzyme in the exo FRET probe detection system. 100 U of exo III enzyme turns to be the best amount and higher amount of the enzyme was not tested because otherwise the volume of the enzyme will be too much in the reaction system.

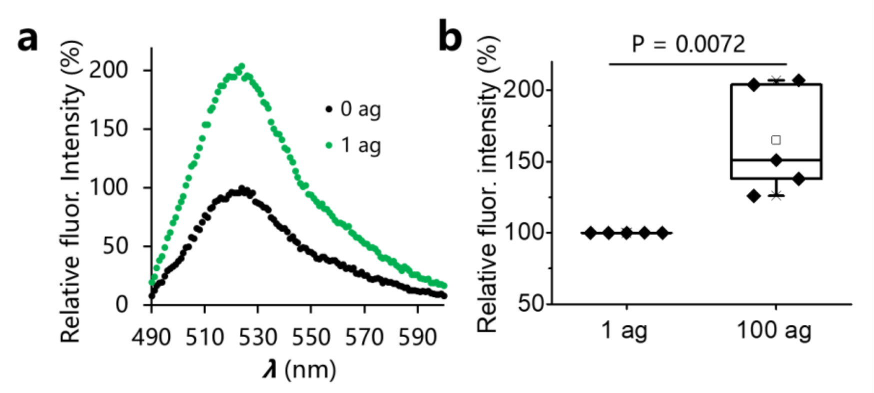

Figure S5. Demonstration of the 1 ag (~4 copies) detection sensitivity for the detection SARS-CoV-2 N-gene using the exo probe and primers. a, A representative detection experiment which shows that the fluorescence (λ_{ex} = 455nm) of the sample reaction containing 1 ag of N-gene RNA shows obviously higher fluorescence intensity than the control reaction (0 ag of N-gene RNA). **b,** Statistical comparison of the relative fluorescence intensities between sample reactions and the control reactions reveals a significant difference between them (n=5 independent experiments, Student's t-test, P=0.0072).

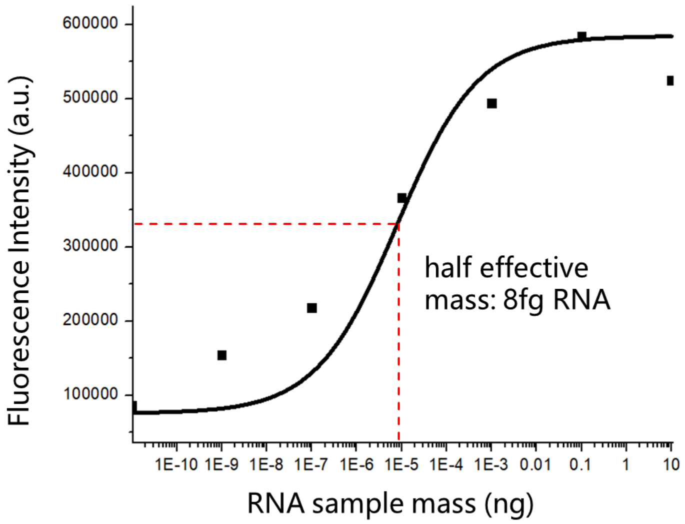

Figure S6. The fluorescence intensities of the samples in the exo FRET probe assay were plotted against RNA sample mass and fitted to a five-parameter Logistic curve. A half effective mass of 8 fg was derived.

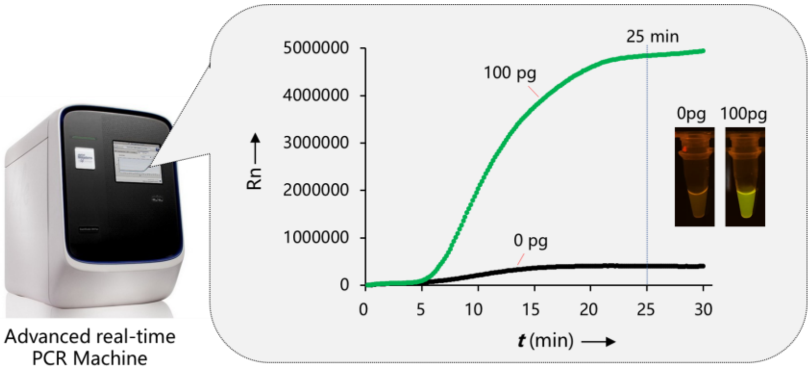

Figure S7. The exo FRET probe can also be implemented in a fully automatic real-time PCR machine which allows real-time determination of the fluorescence intensity of the reaction solution along time (λ_{ex}=470 ± 15 nm, λ_{em}=520 ± 15 nm). It can be seen from the curve that after 25 min, the curve started to reach a plateau, suggesting that 30 min of reaction time in our assay is enough to achieve the maximal fluorescence intensity possible in the exo FRET probe assays. Note that the fluorescence intensity starts to undergo exponential increase after 5 min.

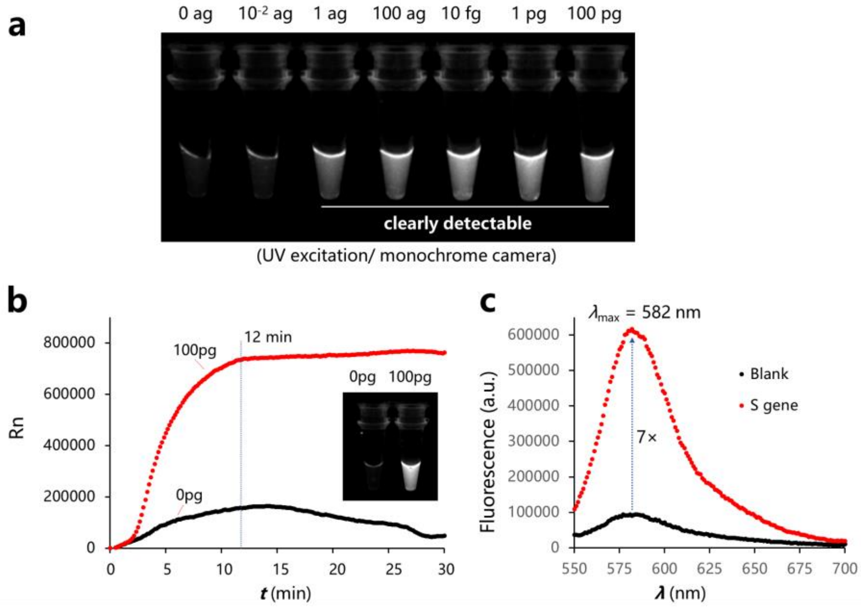

Figure S8. Ultrasensitive detection of S-gene RNA using exo FRET probe. a, The fluorescence readout suggests that as low as 1 ag of RNA can be clearly detected compared to blank control. **b**, Time course of the RT-ERA reaction that is monitored using real-time PCR machine (λ_{ex}=550 ± 11 nm, λ_{em}=586 ± 10 nm). The fluorescence intensity starts to undergo exponential increase after 3 min, which is earlier than in N-gene detection. **c**, After RT-ERA reaction, there is around 7 times of fluorescence enhancement compared to blank control.

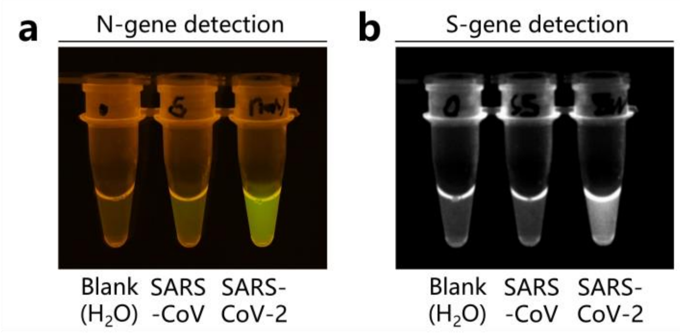

Figure S9. Evaluation of the specificity of exo probes for the detection of SARS-CoV-2 over other highly similar coronavirus variants, herein exemplified by SARS-CoV. a, For the detection of N-gene, only in the presence of SARS-CoV-2 N-gene RNA (100 pg), the RT-ERA reaction turns to be highly fluorescent whereas for the blank reaction (H_2O) or in the presence of SARS-CoV N-gene RNA (100 pg), there is no or only very weak fluorescence. **b**, For the detection of S-gene, only in the presence of SARS-CoV-2 S-gene RNA (100 pg), the RT-ERA reaction turns to be highly fluorescent whereas for the blank reaction (H_2O) or in the presence of SARS-CoV N-gene RNA (100 pg), there is no or only very weak fluorescence.

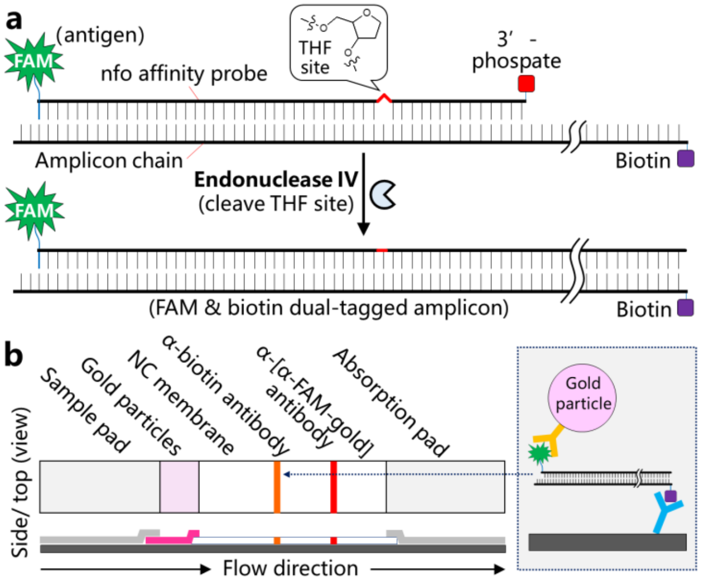

Figure S10. a, The general principle for designing an nfo affinity probe. The nfo probe contains 33 bp in its 5'-side and 15 bp in its 3'-side that can hybridize with the biotin-tagged chain of the amplicon, a 6-FAM moiety (serves as an antigen rather than a fluorophore), a THF site that can be potentially cleaved by Endonuclease IV (Endo IV), and a 3'-phosphate blocking group so that this probe cannot act as a primer. Endo IV cannot cleave the THF site of the probe, but can cleave the THF site when the probe hybridizes with the biotin-tagged amplicon chain in a double-stranded state. After cleavage of the THF site, the 3'-side of the probe is released, converts the nfo probe to a primer that initializes polymerization in the RT-ERA reaction. In the end a FAM and a biotin double-tagged amplicon is generated (down), which could be

easily detected using a lateral flow strip without using any special setups. **b, Schematic view of the principle of lateral flow (LF) strips detection.** The lateral flow strip features a testing line that is immobilized with α-biotin antibody and a control line that is immobilized with an antibody against α-FAM antibody (i.e. α-[α-FAM-gold] antibody). In addition, there is a colloidal gold particle region (the gold particle is coated with α-FAM antibody) below the two lines and above the sample pad. When the sample pad of the test strip is inserted into a RT-ERA reaction solution, the colloidal gold particle will flow along with the eluent and sequentially cross the test and control lines. If the double-tagged amplicon is presented in the RT-ERA reaction, it will bridge the gold particle and the α-biotin antibody in the test line, turns the test line to red (right). Meanwhile the control line that contains α-[α-FAM-gold] antibody shall bind the α-FAM antibody-coated gold particle, suggesting that the LF strip is effective.

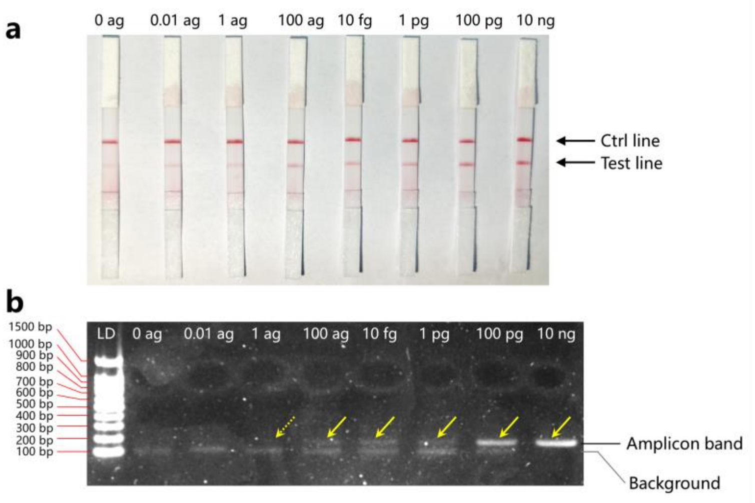

Figure S11. a, another representative LF strip detection results using nfo affinity probe. **b**, 40µl of RT-ERA reaction solution from **a** was subjected to cleanup first and then analyzed via agarose gel electrophoresis (1 %). Clear bands of the expected amplicon (~150 bp) were detected when above 100ag of RNA is detected, and a very faint band of amplicon of 1ag RNA reaction could still be visible. No amplicon band in the blank control reaction (0 ag) could be detected. Note that there is always a background band (M.W. a bit over 100bp) with constant intensity, which could happen in RT-ERA reactions. In addition, the sensitivity in gel electrophoresis is usually lower than that of lateral flow strip detection because the lateral flow strip can enrich bi-functionalized amplicon in a very narrow detection line.

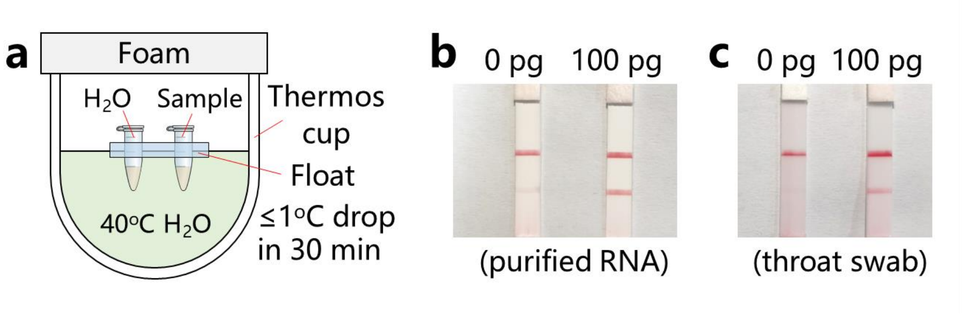

Figure S12. a, A thermos cup can be used as the primary setup for RT-ERA reaction to maintain the temperature. **b**, Results of the LF strips using a thermos cup setup. **c**, RNA specimen in throat swab can be directly used in the detection without any purification.

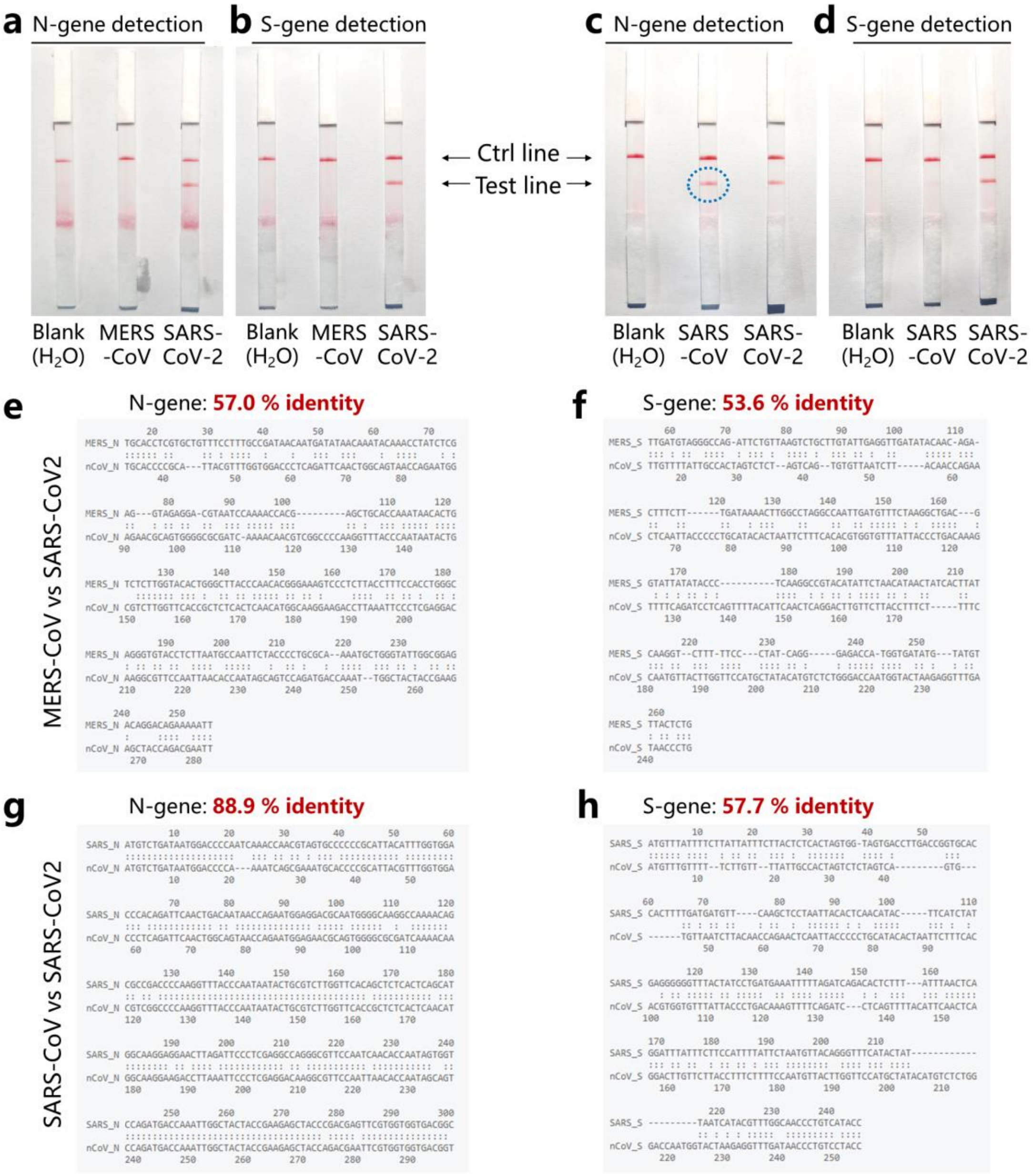

Figure S13. Evaluation of the specificity of nfo probes combined with LF strips for the detection of SARS-CoV-2 over other similar coronavirus variants, herein exemplified by MERS-CoV and SARS-CoV. a,b, For the detection either N- or S-gene, only in the presence of SARS-CoV-2 RNA (100 pg) but not MERS-CoV RNA (100pg), the test line turns red suggesting the specificity against MERS-CoV. **c,d**, For the detection of N-gene, interestingly, in the presence of either SARS-CoV-2 N-gene RNA (100 pg) or SARS-CoV N-gene RNA (100pg), the test line turns red; however, for the detection of S-gene, only in the presence of SARS-CoV-2 S-gene RNA (100 pg) but not SARS-CoV S-gene RNA (100 pg), the test line turns red. These interesting results reveal that exo probe for S-gene can be used to differentiate SARS-CoV-2 from both MERS-CoV and SARS-CoV while exo probe for N-gene can be used to detect either SARS-CoV or SARS-CoV-2 with specificity against MERS-CoV. **e-h**, N- or S-gene sequence identity assessment reveals higher identity between SARS-CoV2 and SARS-CoV than between SARS-CoV2 and MERS-CoV.

Sequence Information

For the following sequence information, customer synthesized gene sequence fragments are featured in capital letters while start and stop codons are highlighted in red.

A. pET-28a(+)_SARS-CoV-2_N plasmid for transcription of SARS-CoV-2 N-gene:
…catatgTCTGATAATGGACCCCAAAATCAGCGAAATGCACCCCGCATTACGTTTGGTGGACCCTCAGATTCAACTG
GCAGTAACCAGAATGGAGAACGCAGTGGGGCGCGATCAAAACAACGTCGGCCCCAAGGTTTACCCAATAATACT
GCGTCTTGGTTCACCGCTCTCACTCAACATGGCAAGGAAGACCTTAAATTCCCTCGAGGACAAGGCGTTCCAATTA
ACACCAATAGCAGTCCAGATGACCAAATTGGCTACTACCGAAGAGCTACCAGACGAATTCGTGGTGGTGACGGTA
AAATGTAAaagctt…

B. pET-28a(+)_SARS-CoV-2_S plasmid for transcription of SARS-CoV-2 S-gene:
…catatgTTTGTTTTTCTTGTTTTATTGCCACTAGTCTCTAGTCAGTGTGTTAATCTTACAACCAGAACTCAATTACCCC
CTGCATACACTAATTCTTTCACACGTGGTGTTTATTACCCTGACAAAGTTTTCAGATCCTCAGTTTTACATTCAACTC
AGGACTTGTTCTTACCTTTCTTTTCCAATGTTACTTGGTTCCATGCTATACATGTCTCTGGGACCAATGGTACTAAGA
GGTTTGATAACCCTGTCCTACCATAAaagctt…

C. pET-28a(+)_SARS-CoV_N plasmid for transcription of SARS-CoV N-gene:
…catatgTCTGATAATGGACCCCAATCAAACCAACGTAGTGCCCCCCGCATTACATTTGGTGGACCCACAGATTCAAC
TGACAATAACCAGAATGGAGGACGCAATGGGGCAAGGCCAAAACAGCGCCGACCCCAAGGTTTACCCAATAATA
CTGCGTCTTGGTTCACAGCTCTCACTCAGCATGGCAAGGAGGAACTTAGATTCCCTCGAGGCCAGGGCGTTCCAAT
CAACACCAATAGTGGTCCAGATGACCAAATTGGCTACTACCGAAGAGCTACCCGACGAGTTCGTGGTGGTGACGG
CAAAATGAAAGAGCTCAGCCCCTAAaagctt…

D. pET-28a(+)_SARS-CoV_S plasmid for transcription of SARS-CoV S-gene:
…catatgTTTATTTTCTTATTATTTCTTACTCTCACTAGTGGTAGTGACCTTGACCGGTGCACCACTTTTGATGATGTTC
AAGCTCCTAATTACACTCAACATACTTCATCTATGAGGGGGGTTTACTATCCTGATGAAATTTTTAGATCAGACACT
CTTTATTTAACTCAGGATTTATTTCTTCCATTTTATTCTAATGTTACAGGGTTTCATACTATTAATCATACGTTTGGCA
ACCCTGTCATACCTTAAaagctt…

E. pET-28a(+)_MERS-CoV_N plasmid for transcription of MERS-CoV N-gene:
…catatgGCATCCCCTGCTGCACCTCGTGCTGTTTCCTTTGCCGATAACAATGATATAACAAATACAAACCTATCTCG
AGGTAGAGGACGTAATCCAAAACCACGAGCTGCACCAAATAACACTGTCTCTTGGTACACTGGGCTTACCCAACAC
GGGAAAGTCCCTCTTACCTTTCCACCTGGGCAGGGTGTACCTCTTAATGCCAATTCTACCCCTGCGCAAAATGCTGG
GTATTGGCGGAGACAGGACAGAAAAATTAATACCGGGAATGGAATTAAGCAACTGGCTCCCAGGTGGTACTTCTA
CTACACTGGAACTGGACCCGAAGCAGCACTCCCATAAaagctt…

F. pET-28a(+)_MERS-CoV_S plasmid for transcription of MERS-CoV S-gene:
…catatgATACACTCAGTGTTTCTACTGATGTTCTTGTTAACACCTACAGAAAGTTACGTTGATGTAGGGCCAGATTC
TGTTAAGTCTGCTTGTATTGAGGTTGATATACAACAGACTTTCTTTGATAAAACTTGGCCTAGGCCAATTGATGTTT
CTAAGGCTGACGGTATTATATACCCTCAAGGCCGTACATATTCTAACATAACTATCACTTATCAAGGTCTTTTTCCCT
ATCAGGGAGACCATGGTGATATGTATGTTTACTCTGCAGGACATGCTACAGGCACAACTCCATAAaagctt…
